# Photoinduced Metal-Free Atom Transfer Radical Polymerization for the Modification of Cellulose with Poly(*N*-isopropylacrylamide) to Create Thermo-Responsive Injectable Hydrogels

**DOI:** 10.3390/ijms25052867

**Published:** 2024-03-01

**Authors:** Xiaohong Liu, Juanli Shen, Ying Wang, Ming Li, Shiyu Fu

**Affiliations:** State Key Laboratory of Pulp and Paper Engineering, South China University of Technology, Guangzhou 510640, China; lxh13424067451@163.com (X.L.); shenjuanli2021@163.com (J.S.); ying91910520@163.com (Y.W.); li163ming@163.com (M.L.)

**Keywords:** metal-free ATRP, UV irradiation, cellulose, injectable hydrogel

## Abstract

Photoinduced metal-free ATRP has been successfully applied to fabricate thermo-responsive cellulose graft copolymer (PNIPAM-*g*-Cell) using 2-bromoisobuturyl bromide-modified cellulose as the macroinitiator. The polymerization of N-isopropylacrylamide (NIPAM) from cellulose was efficiently activated and deactivated with UV irradiation in the presence of an organic-based photo-redox catalyst. Both FTIR and ^13^C NMR analysis confirmed the structural similarity between the obtained PNIPAM-*g*-Cell and that synthesized via traditional ATRP methods. When the concentration of the PNIPAM-*g*-Cell is over 5% in water, it forms an injectable thermos-responsive hydrogel composed of micelles at 37 °C. Since organic photocatalysis is a metal-free ATRP, it overcomes the challenge of transition-metal catalysts remaining in polymer products, making this cellulose-based graft copolymer suitable for biomedical applications. In vitro release studies demonstrated that the hydrogel can continuously release DOX for up to 10 days, and its cytotoxicity indicates that it is highly biocompatible. Based on these findings, this cellulose-based injectable, thermo-responsive drug-loaded hydrogel is suitable for intelligent drug delivery systems.

## 1. Introduction

Cellulose is the most abundant natural polymer in the world. And it has numerous applications in medical fields due to its renewability, biodegradability, recyclability, non-toxicity, biocompatibility, and excellent mechanical properties [1,2]. Additionally, cellulose’s unique feature of having three hydroxyl groups per monomer makes it a distinct platform for modification [3].

Grafting polymerization on a cellulose surface is an effective method of improving the physicochemical properties and enhancing the functional properties of cellulose. Among cellulose graft polymerization technologies, controllable/reactive radical polymerization (CRP) is considered the most effective method of synthesizing graft polymers with well-defined structures [4,5]. Common CRP methods include NMP [6], RAFT [7], and ATRP [8]. Among these techniques, ATRP technology has been effectively utilized to modify cellulose and its derivatives due to its wide range of monomers and initiators [9,10]. However, the disadvantage of traditional ATRP is that the polymerization process requires transition-metal complexes as catalysts, which are not consumed during the polymerization process and are difficult to purify. As a result, metal complexes remain in the product, leading to product aging and other side effects. To address this issue, researchers proposed using ARGET to balance CuI and CuII in ATRP reactions. This method could reduce the Cu concentration from 10,000 ppm to 10 ppm. Nevertheless, residual metal catalysts may contaminate cellulose graft copolymers, limiting their applications in microelectronics and biomaterials.

Organocatalyzed ATRP is a metal-free “grating from” technique that eliminates transition-metal contamination, reducing the toxicity of the graft copolymer. This makes controlled polymerization more practical for application in biological fields such as drug and gene delivery [11]. Recently, the organocatalyzed ATRP method has been effectively utilized in the preparation of graft copolymers [12]. Hawker et al. [13] were the first to report organic catalytic ATRP using PTH as the catalyst, effectively addressing the issue of metal contamination and demonstrating controllable polymerization with a narrow molecular weight distribution. Matyjaszewski et al. [14] conducted metal-free ATRP of acrylonitrile and provided a detailed understanding of the mechanism behind metal-free ATRP. With the development of organocatalyzed ATRP, researchers have begun to apply it to modify cellulose and its derivatives. Lu and colleagues [15] adapted metal-free “grafting from” ATRP to prepare renewable cellulose graft copolymers. They employed 2-bromo-2-phenylacetyl ester-modified ethyl cellulose as a macroinitiator and PTH as a photocatalyst. Chen et al. [16] synthesized CNC-based thermo-responsive fluorescent composite materials using metal-free surface-initiated ATRP technology. However, the preparation of cellulose graft copolymers using organocatalyzed ATRP under homogeneous conditions has not yet been explored, which is essential for its application in the medical field.

**Scheme 1:**
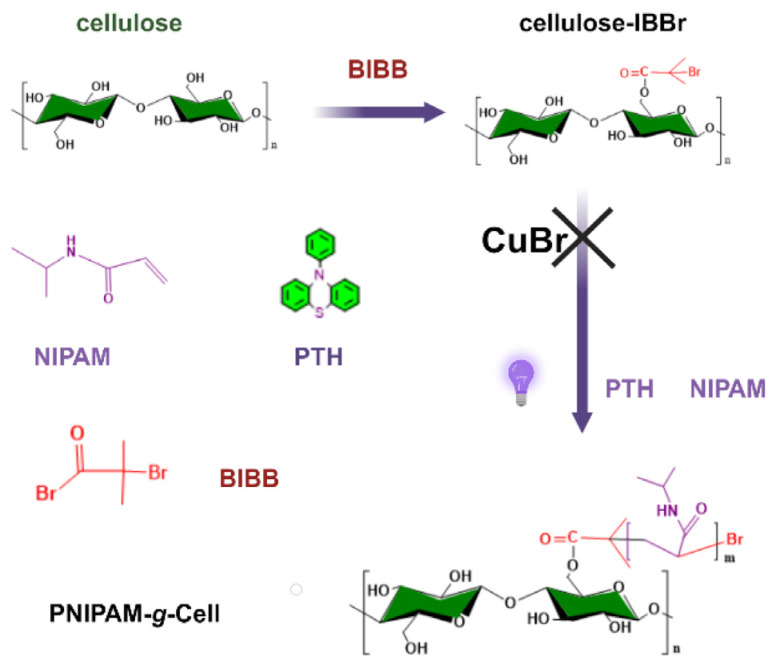
Outlines the preparation of PNIPAM-*g*-Cell. The process begins by replacing the hydroxyl groups on cellulose with BIBB, resulting in cellulose–IBBr. Subsequently, the cellulose backbone is modified by grafting NIPAM using an organocatalyzed ATRP technique.

In our previous work, we utilized copper-catalyzed “grafting from” ATRP to synthesize a cellulose-based injectable thermos-responsive hydrogel loaded with drugs. This material holds great promise for use in intelligent drug delivery systems. However, the presence of CuBr contamination is a concern. To address this issue, we have developed an organocatalyzed ATRP technique to synthesize a cellulose graft copolymer without the use of metals (Scheme 1). We hypothesize that this copolymer will exhibit similar properties as the copper-catalyzed version and can self-assemble into micelles at low concentrations. Furthermore, it has the potential to form a cellulose-based injectable thermo-responsive drug-loaded hydrogel, enabling sustained drug release.

## 2. Results and Discussion

### 2.1. Synthesis and Characterization of PNIPAM-g-Cell

**Figure 1:**
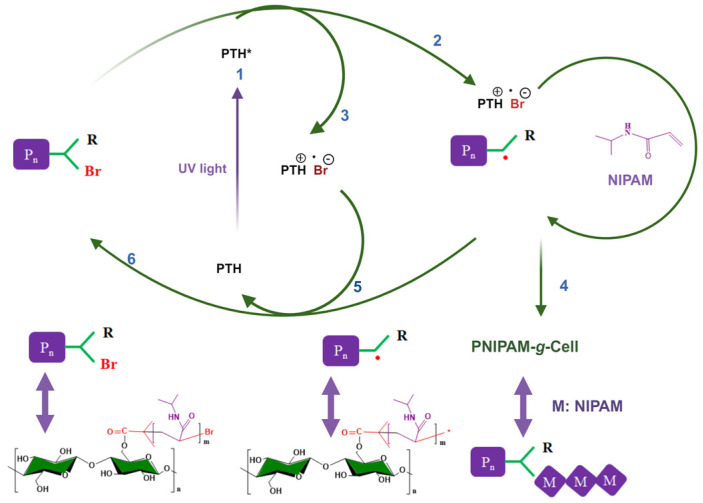
The synthesis mechanism of PNIPAM-*g*-Cell involves the following steps: (1) UV light excites PTH to form the excited-state PTH*; (2) the initiator (with Pn) reacts with the excited-state PTH*; (3) PTH* transfers electrons to Br, regenerating PTH*^+^; (4) the excited-state polymer initiator reacts with monomer NIPAM, leading to chain growth; (5) PTH returns to its ground state; (6) PTH*^+^ is deactivated but can be reactivated by PTH*.

In this study, we attempted to incorporate the BIBB group onto cellulose to create a cellulose macroinitiator for use in organocatalyzed ATRP. The organocatalyzed ATRP reaction was then carried out using the BIBB-functionalized cellulose as the initiator and PTH as the catalyst. The specific mechanism is illustrated in Figure 1. Under UV irradiation, PTH is excited to form a potent reducing agent, PTH*, which generates the oxidation radical cation, PTH*^+^. Subsequently, the initiator (with polymer Pn) is activated to produce free radicals that react with the NIPAM monomer to form an alkyl radical, which is deactivated by PTH*^+^ regenerating the ground state of PTH [17].

**Figure 2:**
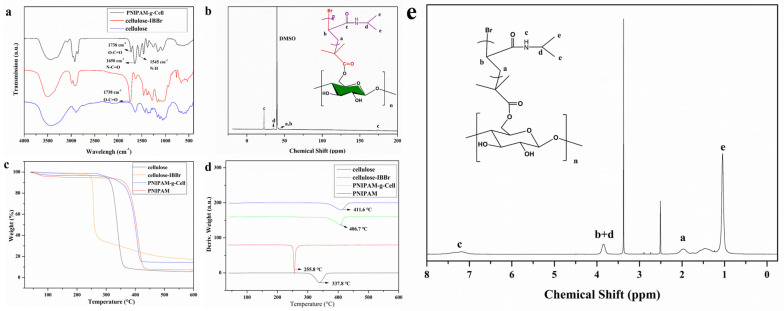
(**a**,**c**) FT-IR spectra (**a**), TG curves (**c**) of cellulose, cellulose–IBBr, and PNIPAM-*g*-Cell. (**d**) DTG curves of cellulose, cellulose–IBBr, PNIPAM-*g*-Cell, and PNIPAM. (**b**,**e**) ^13^C NMR spectrum (**b**), 1H NMR (**e**) of PNIPAM-*g*-Cell.

Figure 2a shows the FT-IR spectra of cellulose, cellulose–IBBr, and PNIPAM-*g*-Cell. A new characteristic absorption peak at 1740 cm^−1^ corresponding to the O–C=O group was observed clearly in the FT-IR spectrum of cellulose–IBBr, indicating the successful attachment of BIBB to cellulose [18]. However, the FT-IR spectrum of cellulose did not exhibit this peak. After the polymerization, strong characteristic absorption peaks at 1650 cm^−1^ corresponding to the N–C=O stretching of amide and 1545 cm^−1^ corresponding to the N-H stretching of amine in the NIPAM repeat units were observed, confirming that the success of the “grafting from” polymerization [18].

The successful preparation of PNIPAM-*g*-Cell was validated through ^13^C NMR spectroscopy and ^1^H NMR spectroscopy. The typical peaks of the side groups of PNIPAM were clearly identified. As demonstrated in Figure 2b, the peak at 174.2 ppm corresponds to the amide carbonyl of PNIPAM (c), while the chemical shifts at 42.3 and 42.5 ppm correspond to the C–C main chain (a, b), the peaks at 34.9 ppm (d) and 22.6 ppm (e) are attributed to the C–N carbon and the CH_3_ carbons, respectively. These results reveal that the cellulose graft copolymer PNIPAM-*g*-Cell was successfully synthesized through organocatalyzed ATRP. The ^1^H NMR spectrum of PNIPAM-*g*-Cell is shown in Figure 2e. The signals of the grafted polyNIPAM are clearly visible and different from those associated with the cellulose backbone, which are much weaker in signal intensity. After this integration, the molar ratio of NIPAM and cellulose was 27.2:1.

The thermal degradation behaviors of cellulose, cellulose–IBBr, and PNIPAM-*g*-Cell were studied using TGA, as depicted in Figure 2c,d. The TGA and DTG curves of cellulose exhibited significant changes after esterification and grafting. The highest temperature at which cellulose decomposed was observed to be 337.8 °C, while that of cellulose–IBBr was 255.8 °C. The lowering of the decomposition temperature of cellulose–IBBr may be a result of the presence of unstable bromine within cellulose–IBBr, which may enhance the rate of decomposition through a free radical mechanism [19]. Conversely, the TGA curve of PNIPAM-*g*-Cell shifted toward higher temperatures, with a peak at 406 °C, which is still slightly lower than that of PNIPAM (411.6 °C) [20]. Moreover, the solid residue of cellulose was 5.2%, and the final solid residue of cellulose–IBBr was 17.2%, which is attributed to the presence of Br in the residual solid after decomposition. Likewise, the final solid residue of PNIPAM-*g*-Cell was also higher than that of PNIPAM due to the presence of Br in the residual solid after the decomposition of PNIPAM-*g*-Cell, which corroborates the existence of cellulose. This shift in decomposition peaks confirmed the successful preparation of PNIPAM-*g*-Cell, and the presence of PNIPAM grafted onto MCC enhanced the thermal stability of the material [21].

### 2.2. The Self-Assembly Behavior of PNIPAM-g-Cell

**Figure 3:**
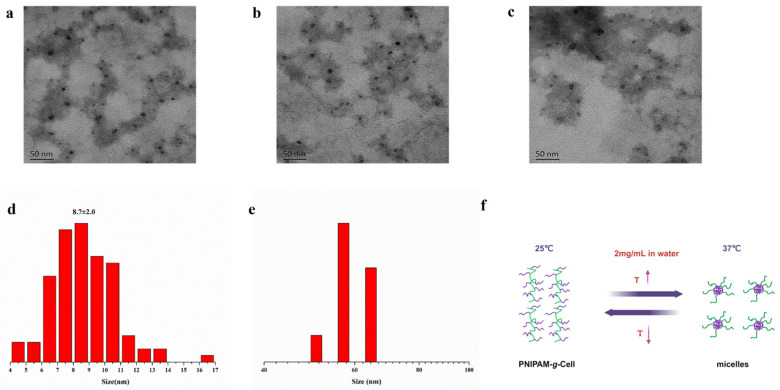
(**a**–**c**) The TEM images of PNIPAM-*g*-Cell micelles in water; (**d**,**e**) the particle size distribution of PNIPAM-*g*-Cell micelles in water obtained through TEM (**d**) and DLS (**e**); (**f**) schematic diagram of self-assembly of PNIPAM-*g*-Cell micelles at 37 °C.

The self-assembly behavior of PNIPAM-*g*-Cell was studied. When the temperature was above the LCST of the PNIPAM-*g*-Cell, it underwent a phase transition to an amphiphilic polymer. At 37 °C (>LCST), the PNIPAM-*g*-Cell formed micelles in water due to its amphiphilic nature. The schematic diagram of the self-assembly process is depicted in Figure 3f. As the temperature increased to 37 °C, the amphiphilic polymer formed spherical micelles in water with a hydrophobic PNIPAM core and a hydrophilic cellulose shell. TEM was used to examine the morphology of these micelles, and the results are presented in Figure 3a–c. The micelles exhibited a uniform spherical morphology with a particle size distribution ranging from 4 to 17 nm, as determined by ImageJ software 1.54 analysis (Figure 3d). The average particle size measured by TEM was 8.7 nm. Additionally, DLS was utilized to determine the size of the micelles, and the results are shown in Figure 3e. The DLS measurements revealed a narrow particle size distribution (50–65 nm) for the PNIPAM-*g*-Cell micelles synthesized via organocatalyzed ATRP technology, which is consistent with the TEM results. However, the average particle size measured by DLS (55.6 nm) was larger than that obtained by TEM, possibly due to differences in measuring wet vs. dry states.

### 2.3. The Sol–Gel Properties and Morphology of PNIPAM-g-Cell Injectable Hydrogels

**Figure 4:**
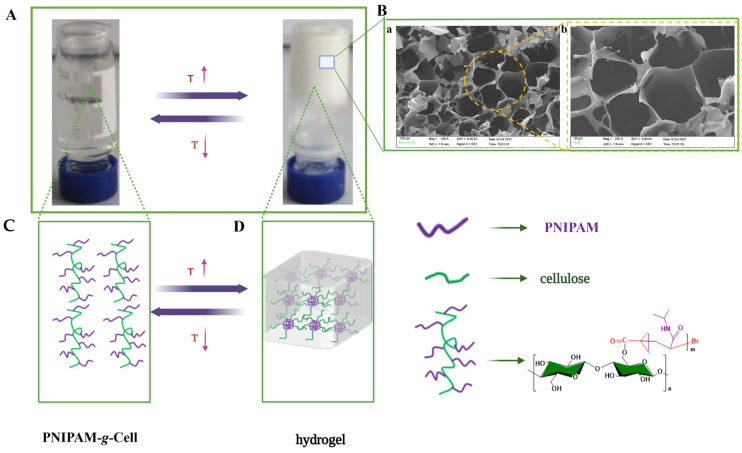
(**A**–**D**) The sol–gel properties (**A**), SEM image (**B**), and the formation mechanism of (**C**,**D**) of the cellulose-based injectable hydrogel obtained from PNIPAM-*g*-Cell.

The sol–gel properties of cellulose-based injectable hydrogels composed of PNIPAM-*g*-Cell were further confirmed through test tube inversion. As demonstrated in Figure 4A, the prepared 5% PNIPAM-*g*-Cell aqueous solution freely flowed within the sample bottle. However, upon stabilization in water at 37 °C for 50 s, the solution became non-flowing. Upon inversion, the solution remained stationary, indicating that PNIPAM-*g*-Cell could form a hydrogel under physiological temperature stimulation (37 °C) within a short period of 50 s. This finding also confirms that the cellulose-based hydrogel composed of PNIPAM-*g*-Cell possesses suitable injectability properties.

The microstructure of the cellulose-based injectable hydrogel composed of PNIPAM-*g*-Cell was examined using scanning electron microscopy (SEM). As depicted in Figure 4B(a,b), the hydrogel exhibited high microporosity and a well-defined three-dimensional network structure. The pores were interconnected, forming an “open-cell” structure with a uniform pore size averaging 100 μm [22]. The formation mechanism of the hydrogel can be attributed to the micellization process, as shown in Figure 4C,D. Specifically, the PNIPAM-*g*-Cell smart-blocks became more hydrophobic when the temperature reached 37 °C, transitioning into an amphiphilic copolymer. PNIPAM-*g*-Cell can form micelles through self-assembly, thanks to the self-assembly properties of amphiphilic polymers. Furthermore, at a concentration of 4.2%, the micelles transformed into inter-molecular micelles, ultimately leading to the formation of a 3D hydrogel network (Figure 4D).

### 2.4. Mechanical Properties of PNIPAM-g-Cell Injectable Hydrogels

**Figure 5:**
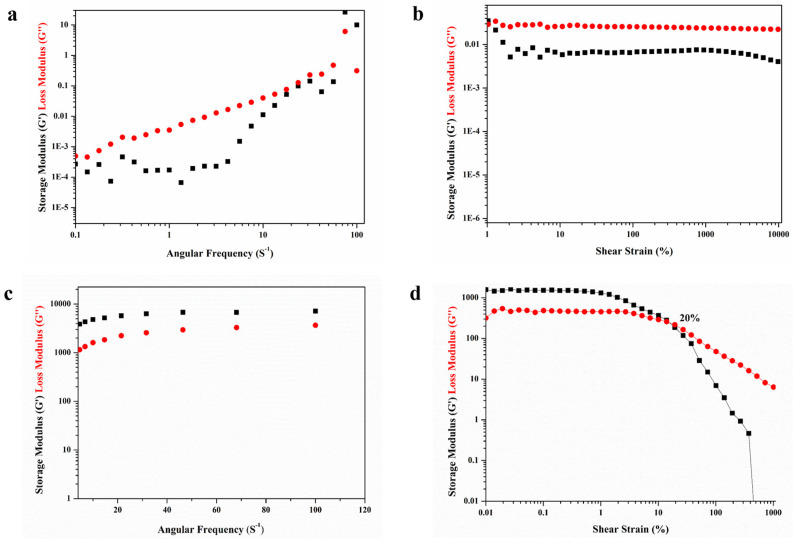
(**a**–**d**) Frequency sweep (**a**) and oscillatory stress sweep analysis (**b**) of the PNIPAM-*g*-Cell at 4 °C. Frequency sweep (**c**) and oscillatory stress sweep analysis (**d**) of the PNIPAM-*g*-Cell hydrogel at 37 °C.

The mechanical properties of injectable cellulose-based hydrogels composed of PNIPAM-*g*-Cell at 37 °C were assessed using rheology, as depicted in Figure 5c,d. The storage and loss moduli as a function of angular frequency (ω) for the PNIPAM-*g*-Cell at 4 °C are depicted in Figure 5a, while the storage and loss moduli as a function of angular frequency (ω) for the PNIPAM-*g*-Cell injectable hydrogel at 37 °C are depicted in Figure 5c. As can be seen in Figure 5a–c, at 4 °C, G′ was less than G″, and at 37 °C, G′ was greater than G″, demonstrating the effect of temperature on PNIPAM-*g*-Cell and exhibiting a sol–gel transition phenomenon. The elastic module of the PNIPAM-*g*-Cell injectable hydrogel was found to be 7062 Pa, which is higher than that reported for other injectable hydrogels or CNC-reinforced hydrogels [23]. This high value indicates that the hydrogel is suitable for use in sustained delivery applications [24].

Furthermore, as the shear stress increased from 1% to 700%, the storage modulus (G′) of injectable cellulose hydrogels decreased gradually. Additionally, when the shear strain exceeded 20%, the storage modulus (G′) decreased to lower than the loss modulus (G″), indicating a transition from a the solid-like to a liquid-like phase. As depicted in Figure 5d, the hydrogel was destroyed at a shear strain value of 20%, which is commonly referred to as the yield strain [25].

### 2.5. In Vitro Release of DOX from PNIPAM-g-Cell Injectable Hydrogels and Their Biocompatibility

**Figure 6:**
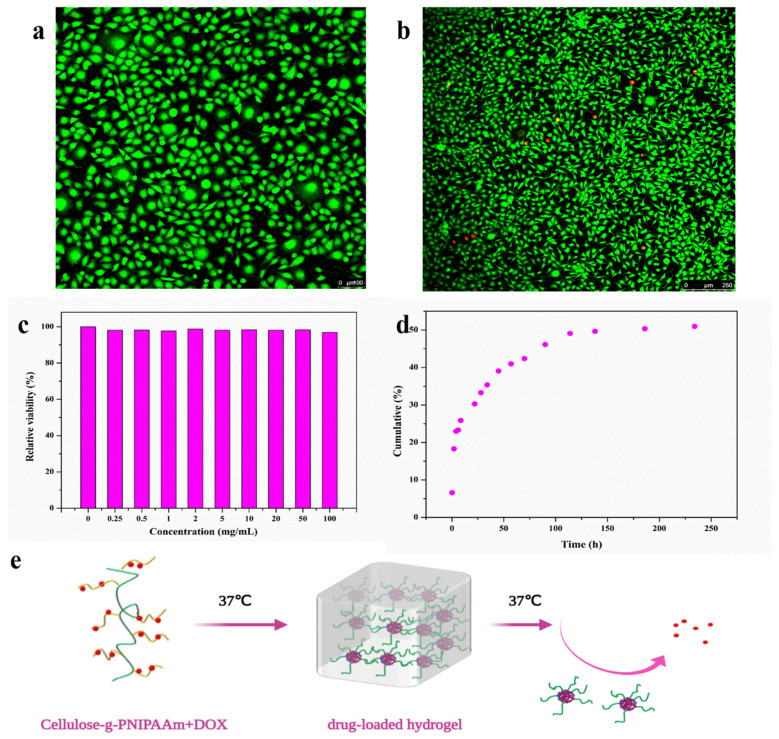
(**a**) Fluorescence image of L929 cells with 100 mg/mL PNIPAM-*g*-Cell injectable hydrogel extract; (**b**) fluorescence images of L929 cells with 5 mg/mL cellulose–*g*-PNIPAAm injectable hydrogel extract; (**c**) viability histograms of L929 cells after co-cultivation with different concentrations of PNIPAM-*g*-Cell injectable hydrogel leaching liquor; (**d**) release curves of DOX in the PNIPAM-*g*-Cell injectable cellulose hydrogel at 37 °C; (**e**) schematic diagram of loading and release mechanism of the PNIPAM-*g*-Cell injectable cellulose hydrogel.

To investigate its drug release behavior, DOX was incorporated into the PNIPAM-*g*-Cell injectable hydrogels. As depicted in Figure 6d, the cumulative drug release from the hydrogels lasted for up to 15 days, which is longer than that of other injectable hydrogels. For instance, Omidi et al. [26] created a pH-responsive injectable hydrogel composed of chitosan (CS), amino-functionalized cellulose nanowhisker (WN), and amino-functionalized graphene oxide (GN). In vitro drug release studies revealed that the drug could be continuously released for approximately 10 h. In contrast, the PNIPAM-*g*-Cell injectable hydrogels prepared in this study could sustainably release DOX for 240 h (10 days). Moreover, the release curve indicated no significant burst release from the hydrogels, suggesting that they possessed a stable release rate and prolonged release time. This phenomenon is closely related to the drug-loading mechanism of the hydrogels, as depicted in Figure 6e. As illustrated, DOX was encapsulated within the hydrophobic core of micelles at 37 °C, followed by a sustained controlled release at 37 °C. Additionally, the hydrogel exhibits good injectability, making it a promising candidate for use in injectable drug delivery systems.

The safety of injectable hydrogels is ensured by their good biocompatibility. In this study, the cytocompatibility of PNIPAM-*g*-Cell was evaluated using the L929 cell live/dead double-staining method. As shown in Figure 6c, the cells exhibited high activity at various concentrations of PNIPAM-*g*-Cell injectable hydrogel extract. Even at a concentration of 100 mg/mL, the survival rate of cells remained as high as 96.9%. Additionally, Figure 6a presents the fluorescence images of live/dead L929 cells cultured in a 100 mg/mL PNIPAM-*g*-Cell injectable hydrogel extract for 24 h. In contrast, Figure 6b shows the fluorescence images of live/dead L929 cells cultured in a 5 mg/mL cellulose–g-PNIPAAm injectable hydrogel extract (prepared using the CuBr/PMDETA system) for 24 h. As seen from the figures, the PNIPAM-*g*-Cell hydrogels prepared using the organic photocatalytic ATRP method described in this study showed almost no red spots (indicative of dead cells) even at a high extract concentration of 100 mg/mL, with green fluorescent spots (indicating living cells) covering nearly the entire field of view. However, the hydrogels prepared using traditional ATRP techniques with CuBr/PMDETA as the catalyst system in previous studies exhibited red fluorescent spots, representing dead cells, at an extract concentration as low as 5 mg/mL. These results demonstrate that the PNIPAM-*g*-Cell injectable hydrogel prepared through organocatalyzed ATRP exhibits excellent biocompatibility, suggesting its potential for application in drug delivery systems.

## 3. Materials and Methods

### 3.1. Materials

MCC (Aladdin), LiCl (Aladdin AR 99%), DMAc (Aladdin AR 99%), BIBB (98%, Aladdin), N,N-Dimethylformamide (DMF, Aladdin 99.5%), and 10-Phenylphenothiazine (PTH, 98%) were purchased from Fuzhou Cangshan Chuxu New material Co., Ltd. in Fuzhou, China. NIPAM (Aladdin 97%) was purified by recrystallization in hexane and dried under vacuum at 40 °C for 24 h.

### 3.2. Characterization

The FT-IR spectra were obtained using a Bruker TENSOR27 Spectrum FT-IR spectrophotometer. The ^13^C NMR spectra of the PNIPAM-*g*-Cell sample were recorded on a AVANCE III HD 600 spectrometer (Bruker Corporation, Karlsruhe, Germany) with DMSO-d6 as the solvent. The X-ray photoelectron spectroscopy (XPS) spectra of the cellulose, cellulose–IBBr, and PNIPAM-*g*-Cell were measured on a Thermo ESCALAB 250XI (Thermo Fisher Scientific, Waltham, MA, USA). TGA was conducted on a PerkinElmer instrument, Shanghai, China. At a rate of 10 °C/min, the samples were heated from room temperature to 600 °C in a dynamic nitrogen atmosphere. TEM images were obtained using an H-7650 (Hitachi, Tokyo, Japan). DLS measurements were carried out using a Nanoparticle analyzer SZ-100Z from HORIBA Corporation, Kyoto, Japan. SEM (Zeiss Merlin, Cambridge, MA, USA) was used to observe the micromorphology of the PNIPAM-*g*-Cell injectable hydrogel.

### 3.3. Methods

#### 3.3.1. The Dissolution of MCC

MCC was dissolved in a LiCl/DMAc system [27]. The procedure involved the following steps: Firstly, MCC (3.24 g, 0.02 mol) and LiCl (9.72 g) were dried in a vacuum oven at 100 °C for 24 h. Then, the dried MCC was added to 95.0 g of DMAc and activated at 140 °C for 2 h. Subsequently, LiCl was added, and the mixture was stirred for an additional 2 h at 100 °C. Finally, it was slowly cooled to room temperature while stirring until dissolution was achieved.

#### 3.3.2. Synthesis of PNIPAM-*g*-Cell Copolymer

Firstly, the cellulose macroinitiator (cellulose–IBBr) was synthesized according to the method reported by Chang et al. [28]. To synthesize the PNIPAM-*g*-Cell copolymer, the following procedure was followed [15]. Firstly, 0.1 g (0.12 mmol of Br) of cellulose–IBBr was dissolved in DMF. Then, 2.78 g (24.5 mmol) of NIPAM and 0.0014 g (0.005 mmol) of PTH were added into the flask under nitrogen protection. The reaction system was degassed through three cycles of freeze–evacuate–thaw to ensure an oxygen-free environment. The mixture was then exposed to ultraviolet light at room temperature for 3 h while being stirred continuously during irradiation. After the completion of the reaction, the solution was poured into deionized water and placed in a dialysis bag for 3 days (with the water being changed every 6 h). The final cellulose graft copolymer (PNIPAM-*g*-Cell) product was obtained through rotary evaporation and freeze drying. The graft ratio and monomer conversion were calculated using Equations (1) and (2), respectively.

(1) $$G=\frac{W_{2}-W_{1}}{W_{1}}\times 100\%$$

where *G* is the graft ratio, *W*_2_ is the dry weight of PNIPAM-*g*-Cell (2.12 g), and *W*_1_ is the dry weight of cellulose–IBBr (0.1 g). According to this calculation, the graft ratio in this article was found to be 2020%.

(2) $$\mathrm{monomer}\;\mathrm{conversion}\;(\%)=\frac{w_{3}-w_{2}}{w_{1}}\times 100\%$$

where *w*_2_ is the dry weight of cellulose–IBBr (0.1 g), *w*_3_ is the dry weight of PNIPAM-*g*-Cell (2.12 g), and *w*_1_ is the mass of the initial added NIPAM (2.78 g). Based on this, the monomer conversion was 73% in this article.

The molecular weight of PNIPAM in the synthesized PNIPAM-*g*-Cell, as determined by a gel permeation chromatograph (GPC), was 28,054 g/mol.

#### 3.3.3. GPC

The molecular weight and distribution of the side chains of the graft copolymers were determined using a GPC from P1100 (Dalian Yili Special Instrument Co., Ltd., Dalian, China), with water as the mobile phase and dextran standards for universal calibration. Samples that were completely dissolved were filtered through a 0.2 µm membrane filter before being injected into the GPC using a microliter syringe to test the polymer’s molecular weight and its distribution. The PNIPAM-*g*-Cell graft copolymers were hydrolyzed before the molecular weight measurement, following the hydrolysis procedure referenced from Lin Chunxiang et al. [29], with specific steps as follows: 0.04 g of PNIPAM-*g*-Cell graft copolymer was added to 20 mL of a 1.5 mol/L hydrochloric acid solution and then placed in an oil bath at 90 °C for 72 h. After the reaction, the mixture was filtered to remove the unhydrolyzed cellulose backbone, and the filtrate was vacuum-dried to obtain PNIPAM.

#### 3.3.4. Sol–Gel Transition

The sol–gel transition of PNIPAM-*g*-Cell was confirmed using the test tube inversion method, which involved the following steps: Firstly, an aqueous solution with a concentration of 50 mg/mL PNIPAM-*g*-Cell was prepared at a low temperature. The solution was then transferred to a 10 mL centrifuge tube. The tube was then placed in a 37 °C water bath for approximately 2 min. Finally, the centrifuge tube was inverted to demonstrate the successful conversion of sol–gel.

#### 3.3.5. The In Vitro Study of the Release of DOX from PNIPAM-*g*-Cell Injectable Thermos-Responsive Hydrogel

The experimental steps for the in vitro release of DOX from PNIPAM-*g*-Cell thermosensitive hydrogel were described in previous research [30]. The concentration of DOX used in the release medium was determined by measuring its absorbance value at 481 nm using a UV/Vis spectrophotometer. To obtain the standard curve, DOX·HCl solutions with concentrations ranging from 6.25 × 10^−4^ mg/mL to 0.02 mg/mL were prepared in PBS. The UV/Vis spectrophotometer absorbance value at 481 nm was taken for each concentration to generate the standard curve (3):

(3) y=0.0502x−0.000242 (r=0.999)

The drug release percentage was calculated using the formula below:

(4) $$\mathrm{cumulative}\;\mathrm{release}\;(\%)=M_{t}/M_{0}\times 100$$

The amount of DOX released from the hydrogels at time *t* is represented by *M_t_*, while *M*_0_ denotes the initial amount of DOX loaded into the hydrogel.

#### 3.3.6. The Mechanical Properties of the Injectable Hydrogel Composed of PNIPAM-*g*-Cell

The rotary rheometer (MCR 302) was used to assess the mechanical properties of the PNIPAM-*g*-Cell injectable hydrogel. The copolymer was formed into a 50 mg/mL aqueous suspension prior to testing. The test conditions involved sweeping the oscillatory strain at a fixed frequency of 1 Hz at 37 °C or 4 °C, with the strain increasing logarithmically from 0.01% to 1000%. Frequency sweeps were then performed on the hydrogel at a strain of 1% (linear viscoelastic zone) and an oscillation frequency of 0.01~100 s^−1^ at 37 °C or 4 °C.

#### 3.3.7. The Cytocompatibility of the Cellulose-Based Injectable Thermo-Responsive Hydrogel

The cytotoxicity of the PNIPAM-*g*-Cell injectable hydrogel was evaluated using the CCK-8 method and Calcein-AM/PI staining method. The specific operation steps were consistent with those described in our previous work [31].

## 4. Conclusions

We successfully modified cellulose under homogeneous conditions using organocatalyzed ATRP, a thermos-responsive cellulose graft copolymer (PNIPAM-*g*-Cell) successfully fabricated by using 2-bromoisobuturyl bromide-modified cellulose as the macroinitiator and PTH as the catalyst under ultraviolet light. FTIR and ^13^C NMR confirmed that the synthesized cellulose graft copolymers have the same structure as those obtained through traditional ATRP. At concentrations above 5% in water, PNIPAM-*g*-Cell can form an injectable thermos-responsive hydrogel composed of micelles at 37 °C. Rheological experiments indicated that PNIPAM-*g*-Cell exhibits distinct rheological properties at 4 °C and 37 °C, confirming the sol–gel transition characteristics of PNIPAM-*g*-Cell. The PNIPAM-*g*-Cell injectable hydrogel could sustain the release of DOX for up to 10 days, and cytotoxicity experiments showed that the PNIPAM-*g*-Cell injectable hydrogel prepared through organocatalyzed ATRP displays superior biocompatibility compared with the CuBr/PMDETA system. Furthermore, since organic photocatalysis is a metal-free ATRP method, it eliminates the challenge of transition-metal catalysts remaining in the final product, making this cellulose-based graft copolymer a promising material for biomedical applications.

## Data Availability

Data are contained within the article.

## Abbreviations

RAFT	Reversible addition–fragmentation chain transfer polymerization
BIBB	2-Bromoisobuturyl bromide
ARGET	Activators regenerated by electron transfer
NMP	Nitroxide-mediated polymerization
MCC	Microcrystalline cellulose
LiCl	Anhydrous lithium chloride
DMAc	N,N-dimethylacetamide
NIPAM	N-isopropylarcylamide
DMSO	Dimethylsulfoxide
PBS	Phosphate-buffered solution
DOX	Doxorubicin
LCST	Lower critical solution temperature
ATRP	Atom transfer radical polymerization
FTIR	Fourier transform infrared spectroscopy
TEM	Transmission electron microscope
SEM	Scanning electron microscope
DLS	Dynamic light scattering
TGA	Thermogravimetric analysis
